# Predicting lung metastasis after radical colorectal cancer surgery: a two center retrospective cohort study and nomogram development

**DOI:** 10.3389/fonc.2025.1733538

**Published:** 2026-01-12

**Authors:** Yun Liang, Zichang Ma, Siquan Zhong, Zheng zheng, Ming He, Junjie Lu, Yile Liu, Chaolun Deng, Jiangtao Huang, Zehui Liu, Rui Ding, Zheng Chen, Chunzai Feng, Yonghui Su

**Affiliations:** 1 Department of Gastrointestinal Surgery, The Fifth Affiliated Hospital of Sun Yat-sen University, Zhuhai, Guangdong, China; 2 Division of Gastrointestinal Surgery Center, First Affiliated Hospital of Sun Yat-sen University, Guangzhou, China; 3 Department of Radiology, The Fifth Affiliated Hospital of Sun Yat-sen University, Zhuhai, Guangdong, China; 4 Department of surgical oncology, Zhongshan City People’s Hospital, Zhongshan, Guangdong, China

**Keywords:** colorectal cancer, prediction nomogram, pulmonary metastases, radical surgery, survival prognosis

## Abstract

**Background:**

Colorectal cancer (CRC) is a leading cause of cancer-related death, with distant metastasis being the primary driver of mortality. Lung metastases are among the most frequent sites of extra-abdominal metastasis in CRC patients. Early identification of high-risk patients for pulmonary metastasis after radical surgery is critical for timely intervention and potentially improved patient outcomes.

**Methods:**

We analyzed a cohort of 399 CRC patients who underwent radical surgery and followed their postoperative imaging outcomes. We developed a predictive model using univariate and multivariate logistic regression analyses based on clinical and pathological characteristics. Patients were stratified into two groups based on the median value of the logistic regression-derived risk score. Subsequently, Kaplan-Meier survival analysis was performed to compare survival curves between the two groups.

**Results:**

The predictive model incorporated four independent factors: carcinoembryonic antigen (CEA), N stage, perineural invasion, and surgical approach. The nomogram demonstrated strong discrimination and calibration in both the training and validation cohorts. The training cohort achieved an area under the curve (AUC) of 0.785 (95% CI: 0.725-0.845), while the validation set demonstrated an AUC of 0.779 (95% CI: 0.696-0.863). The sensitivity and specificity were 73.3% and 71.8%, respectively. LMFS was significantly different (P < 0.001) in both based on the model scores. Decision Curve Analysis (DCA) indicated the potential clinical utility of the model.

**Conclusion:**

Our predictive model can assist clinicians in identifying high-risk patients prone to developing pulmonary metastasis following radical resection of colorectal cancer. This facilitates personalized treatment strategies, potentially leading to improved prognosis for CRC patients.

## Introduction

As a major global health challenge, colorectal cancer (CRC) ranks third in incidence and is the second leading cause of cancer deaths worldwide (1). While multimodal treatment strategies have reduced local recurrence rates, high rates of distant metastasis remain a significant challenge, leading to a need for improved overall survival.

Distant metastasis is the primary cause of death in CRC patients, with approximately 20% of patients presenting with distant disease at diagnosis and about one-third developing metastasis after curative surgery (2, 3). Recurrence typically occurs within three years, with distant metastases frequently observed in the liver, lungs, and peritoneum (4). The prognosis for metastatic colorectal cancer (mCRC) remains exceedingly poor, with a 5-year survival rate of less than 20% in the absence of medical intervention (5).

In colorectal cancer, the lung is the second most common site of distant metastasis after the liver, and the most frequent extra-abdominal site. Although timely radical resection combined with systemic chemotherapy improves survival, a substantial proportion of patients still develop pulmonary recurrence after curative surgery (6, 7). Data from the PulMiCC program reveal that the natural history of CRC lung metastases is less aggressive than previously thought, with 5-year survival rates exceeding the historically dismal rate of approximately 0% without oncological intervention (8, 9), These findings highlight the biological and prognostic differences between pulmonary and other visceral metastases (e.g., hepatic or peritoneal), underscoring the need for dedicated risk-stratification tools to estimate individual postoperative recurrence risk. Multimodal treatment initiated upon radiological detection—including perioperative chemotherapy, pulmonary metastatic lesion resection, stereotactic body radiotherapy (SBRT), or percutaneous thermal ablation—can achieve 5-year survival rates of 20–70% (10–13). Therefore, early identification of patients at high risk for pulmonary metastasis following radical resection could help improve survival outcomes and promote personalized approaches in cancer treatment (14–16).

While nomograms have proven valuable in predicting risk and prognosis for various cancers (17, 18), there is a lack of tools specifically designed to predict lung metastasis in CRC patients following radical surgery.

This study aims to develop a predictive model for identifying high-risk factors associated with lung metastasis in CRC patients following radical surgery. By forecasting the risk of lung metastasis, this model would assist clinicians in prognostication and the development of personalized follow-up and treatment strategies for CRC patients after surgery.

## Methods

### Retrospective study design and population

A comprehensive review and analysis of electronic medical records was conducted on 692 colorectal cancer (CRC) patients treated at the Fifth Affiliated Hospital of Sun Yat-sen University and Zhongshan City People’s Hospital between January 2017 and December 2021. Clinical data, including demographic information (gender, age), the last preoperative blood test results (carcinoembryonic antigen [CEA], carbohydrate antigen 19–9 [CA19-9], carbohydrate antigen 125 [CA125]), primary tumor location, surgical details (surgical method, presence of stoma), and postoperative pathological data (maximum tumor diameter, vascular invasion, perineural invasion, T stage, N stage, TNM stage, MMR status, and Ki67 level), and postoperative chemotherapy, were collected for all patients. Pathologic staging of the tumor was conducted using the 8th edition TNM staging system from the Union for International Cancer Control. Based on the research suggestions by Luchini et al (19), MSI status was divided into MSI (MSI-High) and microsatellite stability (MSS, including MS-Low and MS-Stable). Patients with CRC were classified into two groups: the MSI group (negative staining observed for any one of the MMR proteins) and the MSS group (positive staining for all four MMR proteins). The primary tumor location was identified using colonoscopy and abdominal enhanced computed tomography (CT).

Patients were excluded from the study if they met any of the following criteria: (I) Synchronous metastasis diagnosed before resection, (II) History of other malignancies in the past 5 years, (III) Preoperative neoadjuvant chemotherapy, (IV) Lack of follow-up data or regular chest CT, (V) Undefined pulmonary metastases, (VI) Extrapulmonary organ metastasis without pulmonary metastases by the end of follow-up, or (VII) Missing clinical data. A total of 399 patients met the inclusion criteria and were included in the final study.

**Figure 1:**
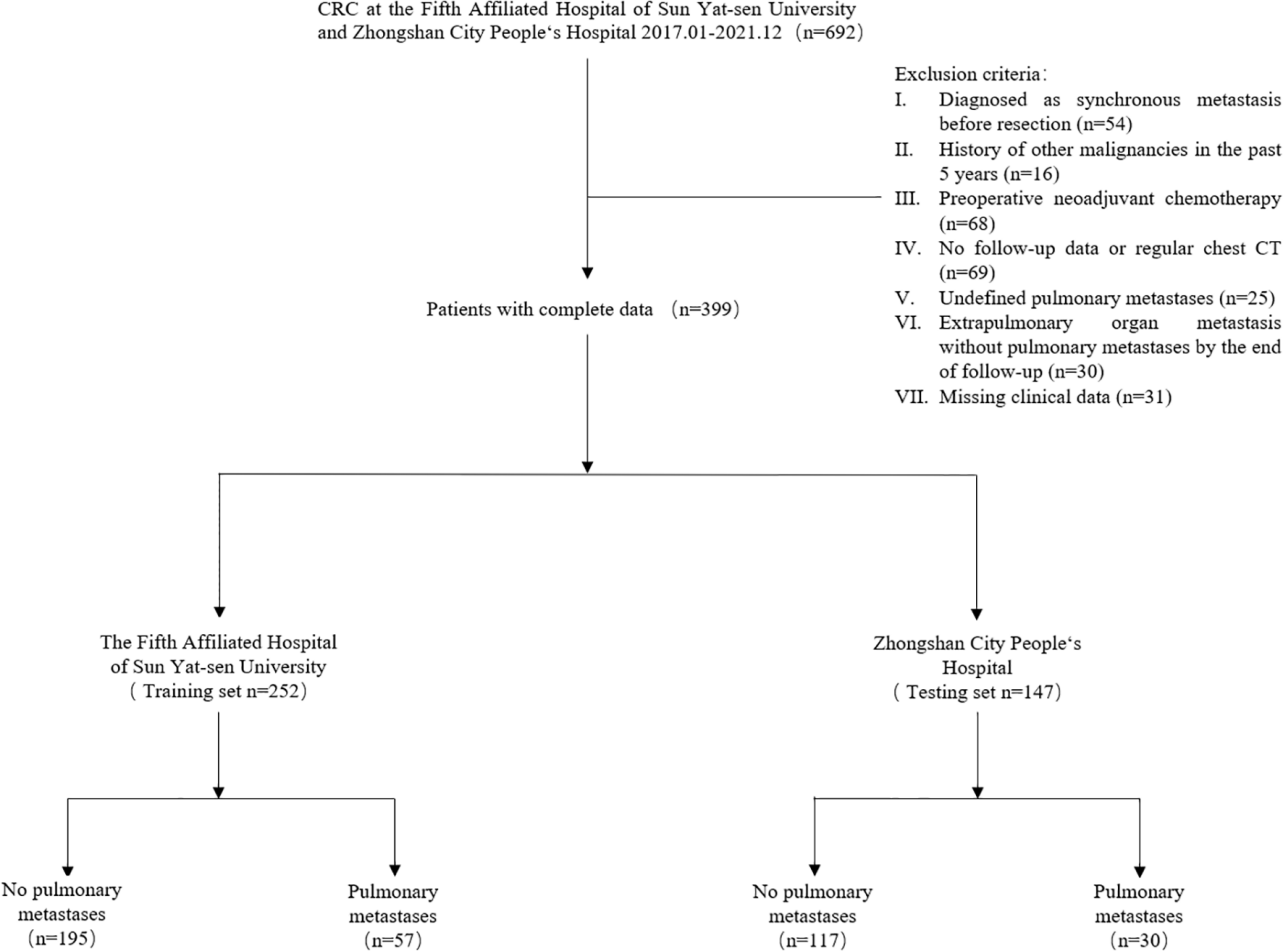
Flowchart of patient selection and exclusion process.

Patient data from the Fifth Affiliated Hospital of Sun Yat-sen University were designated as the training cohort, while data from Zhongshan City People’s Hospital will serve as the testing cohort. Logistic regression analysis was performed on the training cohort to develop a predictive model. This model was then externally validated using the testing cohort. The patient recruitment methodology is illustrated in **Figure 1**. This retrospective study has received approval from the institutional review boards of both the Fifth Affiliated Hospital of Sun Yat-sen University (Ethics Approval Ref. No. K263-1) and Zhongshan City People’s Hospital (Ethics Approval Ref. No. 2024-118). Informed consent from patients has been waived for this study.

### Follow up

Following surgery, all patients underwent regular follow-up assessments including chest and abdominal CT or PET-CT scans. Owing to incomplete follow-up data, the specific details of the postoperative chemotherapy regimens could not be ascertained; therefore, patients who received at least one course were classified into the postoperative chemotherapy group. Follow-up was discontinued for patients who developed lung metastasis detected via these imaging methods. The remaining patients would be monitored until June 2024, with final chest and abdominal CT scan results being documented at that time.

Based on follow-up outcomes, patients were categorized into a lung metastasis group and a non-lung metastasis group. The diagnosis of lung metastasis would be determined based on findings from chest and abdominal CT and PET-CT scans, with the imaging results being independently evaluated by at least two experienced radiologists.

### Study variables

This study collected comprehensive data from electronic medical records, including patient demographics, preoperative blood test results, surgical details, postoperative pathological findings, and follow-up information. Patient characteristics included gender, age, and history of other malignancies. Preoperative blood tests recorded CEA, CA19-9, and CA125 levels. Surgical data encompassed the surgical method, the presence of a stoma, and detailed pathological findings. Postoperative follow-up included documentation of adjuvant chemotherapy status and regular chest and abdominal CT and PET-CT scans, with all imaging reports reviewed by professional radiologists.

### Development and assessment of the nomogram

A logistic regression algorithm was used to assess predictive factors for lung metastasis in the training set, incorporating patient characteristics, laboratory test results, and tumor pathology. Variables that showed statistical significance (P<0.05) in the univariate analysis were incorporated into the following multivariate logistic regression analysis (20). Factors (P<0.05) were found to be independent risk factors for the development of pulmonary metastasis in the multivariate analysis. These factors were then selected to construct a predictive model.

Based on the results of the multivariate logistic regression analysis, a clinical pathological nomogram prediction model was developed. Patients were divided into high-score and low-score groups based on the median value of the logistic prediction in the outcome cohort. The log-rank test was used to analyze LMFS.

The constructed model’s recognition and calibration performance was evaluated using the training set data as an external validation dataset. The area under the curve (AUC) of the Receiver Operating Characteristic (ROC) was used to evaluate the model’s discriminative ability. Calibration curves and the Hosmer-Lemeshow test were employed to evaluate the goodness of fit of the model. Finally, decision curve analysis (DCA) was conducted to assess the clinical utility of the nomogram by calculating the net benefit at different threshold probabilities.

### Validation of the predict model

External validation of the prediction model was performed using data from Zhongshan City People’s Hospital. The logistic regression formula developed in the training set was applied to all patients in the validation set. Subsequently, the performance of the nomogram was evaluated by assessing its calibration (using calibration curves), discrimination (through AUC), and clinical utility (through DCA).

### Statistical analysis

All statistical analyses were conducted using the R statistical software, version 3.5.1 (R Foundation for Statistical Computing; https://www.r-project.org/). Continuous variables were compared using the Mann–Whitney U test, while categorical variables were compared using the chi-square test. Survival analyses were undertaken using Kaplan–Meier analysis. The log-rank test was used for univariate comparisons. A significance level of P< 0.05 was considered statistically significant. Logistic regression analysis, nomogram development, calibration plots, ROC curve analysis, the Hosmer-Lemeshow test, and decision curve analysis (DCA) were performed using the respective R packages. All statistical tests were two-tailed, and P< 0.05 was considered statistically significant.

## Results

### General characteristics

A total of 399 patients were enrolled in this study. Of these, 252 patients from the first medical center were allocated to the training set, while 147 patients from a different medical center constituted the testing set. **Table 1** provides a summary of the distribution of the two datasets, detailing parameters such as patient characteristics and pathological features.

**Table 1:** Comparison between the training cohort and testing cohort.

Characteristic	Training cohort (n=252)	Testing cohort(n=147)	*P*
Gender			0.063
Male	154(61.1%)	75(51.0%)	
Female	98(38.9%)	72(49.0%)	
Age, years	61.5(52-70)	66(55-71)	0.125
Operation			0.028
Laparoscopic	238(94.4%)	146(99.3%)	
Open surgery	14(5.6%)	1(0.7%)	
Stoma			< 0.001
Yes	146(57.9%)	19(12.9%)	
No	106(42.1%)	128(87.1%)	
CEA	3.87(1.91- 9.84)	2.40(1.50 - 4.60)	< 0.001
CA19-9	8.54(3.94 - 16.28)	11.40(6.10 - 20.60)	0.01
CA125	9.86(6.90 - 14.20)	7.80(5.60 - 12.00)	0.001
T stage			0.67
1	24(9.5%)	11(7.5%)	
2	56(22.2%)	30(20.4%)	
3	148(58.7%)	95(64.6%)	
4	24(9.5%)	11(7.5%)	
N stage			0.029
N0	109(43.3%)	81(55.1%)	
N1-N2	143(56.7%)	66(44.9%)	
TNM			0.449
1	54(21.4%)	28(19%)	
2	86(34.1%)	38(25.9%)	
3	112(44.5%)	81(55.1%)	
Primary tumor site			0.399
Colon	106(42.1%)	69(46.9%)	
Rectum	146(57.9%)	78(53.1%)	
Tumor size	4.00(3.00 - 5.00)	3.50(2.50 - 4.50)	0.036
Perineural invasion			0.206
Positive	78(31.0%)	36(24.5%)	
Negative	174(69.0%)	111(75.5%)	
Vascular invasion			< 0.001
Positive	66(26.2%)	63(42.9%)	
Negative	186(73.8%)	84(57.1%)	
MSI status			0.001
MSI	13(5.2%)	5(3.4%)	
MSS	239(94.8%)	142(96.6)	
Ki67	70.00(60.00 - 80.00)	70.00(60.00 - 75.00)	< 0.001
Adjuvant chemotherapy			0.823
Positive	126(50.0%)	71(48.3%)	
Negative	126(50.0%)	76(51.7%)	
Pulmonary metastases			0.696
Positive	57(22.6%)	30(20.4%)	
Negative	195(77.4%)	117(79.6%)	
Time to pulmonary metastasis	31.00(24.00 - 43.00)	29.00(23.00 - 35.00)	0.005

TNM, tumor-node-metastasis; MSI, microsatellite; MSS, microsatellite stability. Categorical data is presented as percentages, while continuous data is presented as median (IQR). Time to pulmonary metastasis refers precisely to the duration from the date of radical CRC surgery to the date when pulmonary metastases were first detected.

In both the training and validation cohorts, the incidence of postoperative pulmonary metastasis in patients with colorectal cancer was comparable (P = 0.696), with observed rates of 22.6% and 20.4%, respectively.

### Screening for predictive factors

In the training set, a univariate analysis was conducted to compare the differences between the lung metastasis group and the non-lung metastasis group. The results of this analysis are presented in **Table 2**. The analysis revealed no statistically significant differences between the two groups with respect to age, gender, stoma status, CA125 levels, T stage, pathological TNM stage, primary tumor location, tumor size, mismatch repair (MMR) status, Ki67 expression, and administration of postoperative chemotherapy (P>0.05).

**Table 2:** Univariate and multivariate logistic regression analysis of the predictors for pulmonary metastasis in training cohort (n=252).

Variable	Univariate analysis	*P*	Multivariate analysis	*P*
	OR (95% CI)		OR (95% CI)	
Gender	0.92(0.51-1.69)	0.797		
Age(years)	1.02(1.00-1.05)	0.518		
Operation	0.27(0.09-0.79)	0.018	0.19(0.05-0.69)	0.011
Stoma	0.58(0.32-1.04)	0.068		
CEA	1.01(1.00-1.02)	0.003	1.01(1.00-1.02)	0.045
CA19-9	1.01(1.00-1.01)	0.019	1.00(1.00-1.01)	0.127
CA125	1.01(1.00-1.03)	0.1		
T stage	2.76(0.31-24.26)	0.356		
N stage	6.00(3.07-11.76)	< 0.001	5.34(2.48-11.49)	< 0.001
Primary tumor site	1.10(0.610-2.00)	0.766		
Tumor size	1.08(0.92-1.27)	0.336		
Perineural invasion	2.8(1.52-5.16)	< 0.001	2.13(1.01-4.48)	0.047
Vascular invasion	2.16(1.15-4.05)	0.017	0.76(0.34-1.71)	0.509
MSI status	0.27(0.03-2.14)	0.216		
Ki67	0.99(0.97-1.01)	0.305		
Adjuvant chemotherapy	1.51(0.83-2.74)	0.177		

Compared to the cohort without lung metastasis, the group with lung metastasis exhibited a significantly higher proportion of individuals who underwent open surgery during radical procedures (P = 0.018). Additionally, in the final preoperative blood sample, patients with lung metastasis demonstrated markedly elevated levels of CEA (P = 0.003) and CA19-9 (P = 0.019) relative to those without lung metastasis.

In the pathological assessment of tumors, patients exhibiting lymph node invasion (N1-2) (P<0.001), perineural invasion (P<0.001), and vascular invasion (P = 0.017) demonstrated a higher propensity for developing lung metastasis postoperatively. Subsequent multivariate logistic regression analysis of these variables revealed that open surgery, CEA levels, N stage, and perineural invasion are significantly associated with the incidence of postoperative lung metastasis in colorectal cancer. These factors may serve as independent predictors for lung metastasis.

### Risk prediction nomogram construction and performance assessment

**Figure 2:**
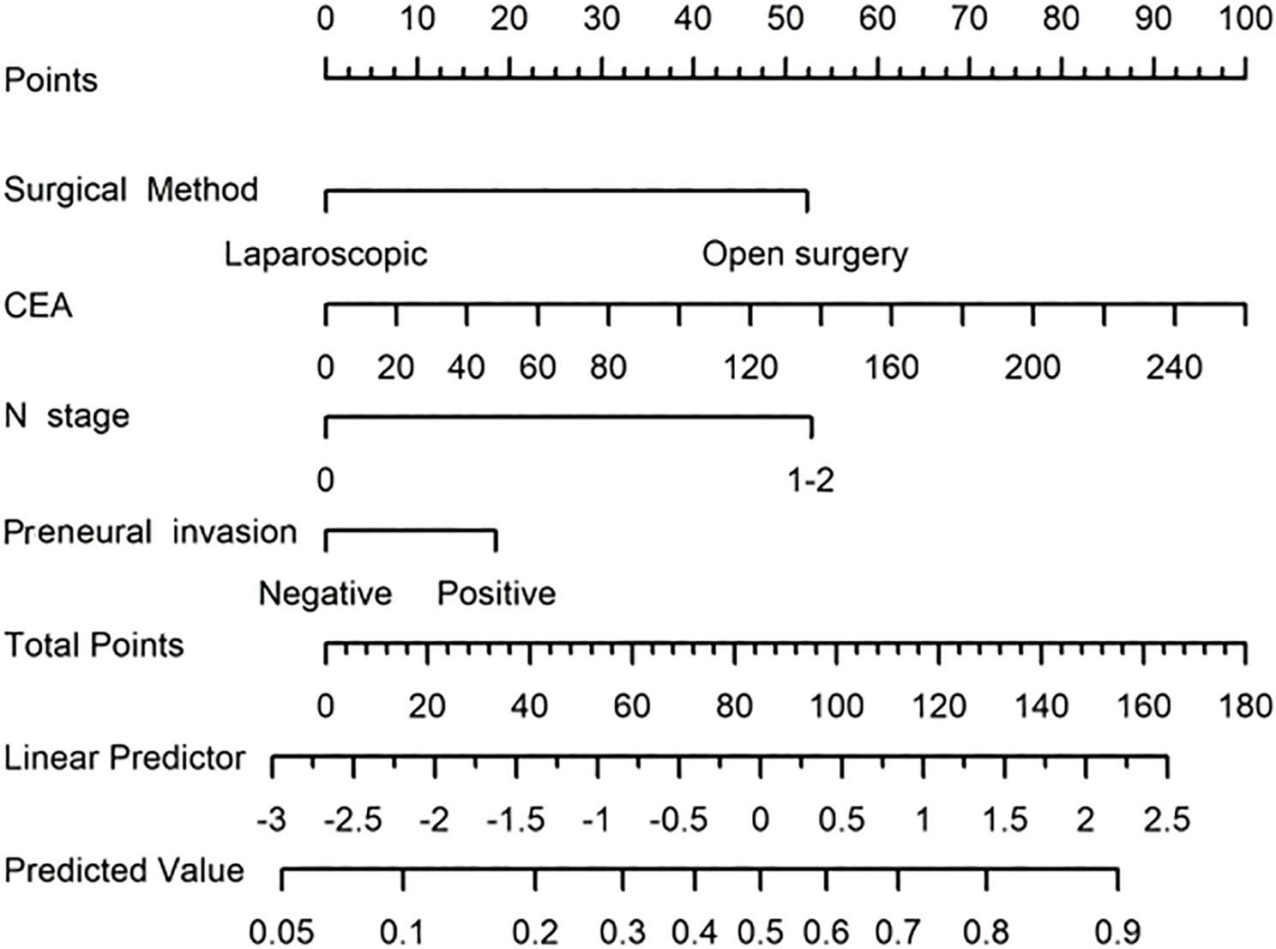
Clinic-pathological nomogram for predicting the probability of lung metastasis in CRC patients following radical surgery. (To use the nomogram, locate your patient’s risk factors, draw lines from their values to the “Points” axis, sum the points, then draw a line down from the total points to find the predicted outcome on the “Predicted Value” axis).

**Figure 3:**
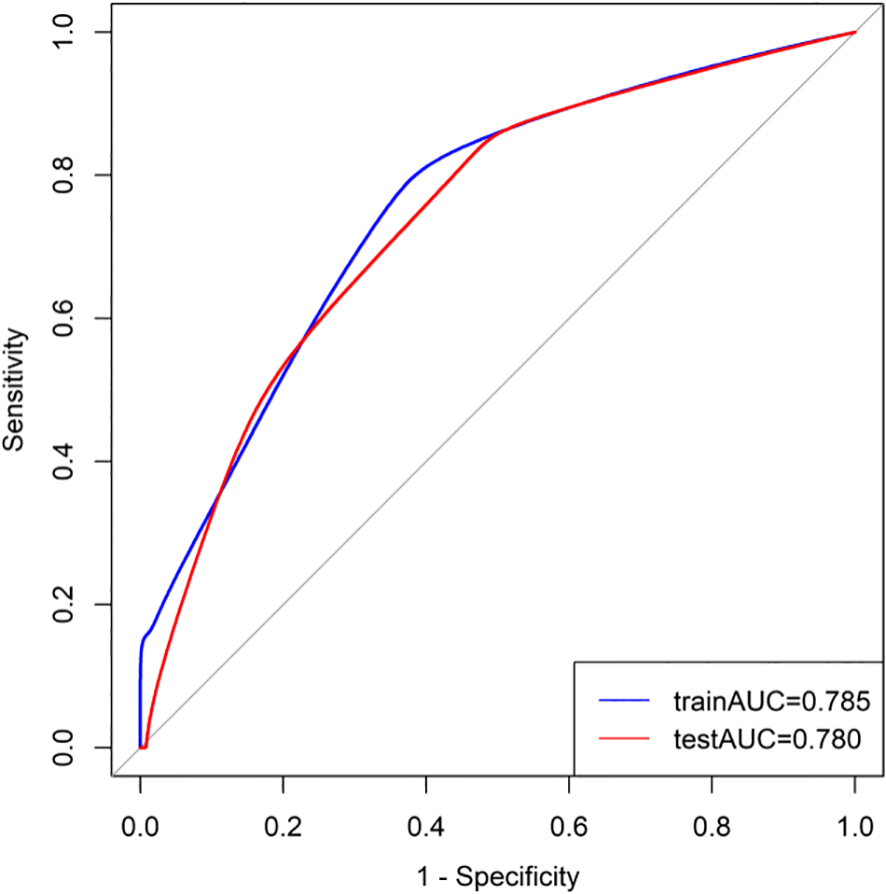
Receiver operator characteristic curve of the clinic-pathological nomogram in the training set and testing set.

**Figure 4:**
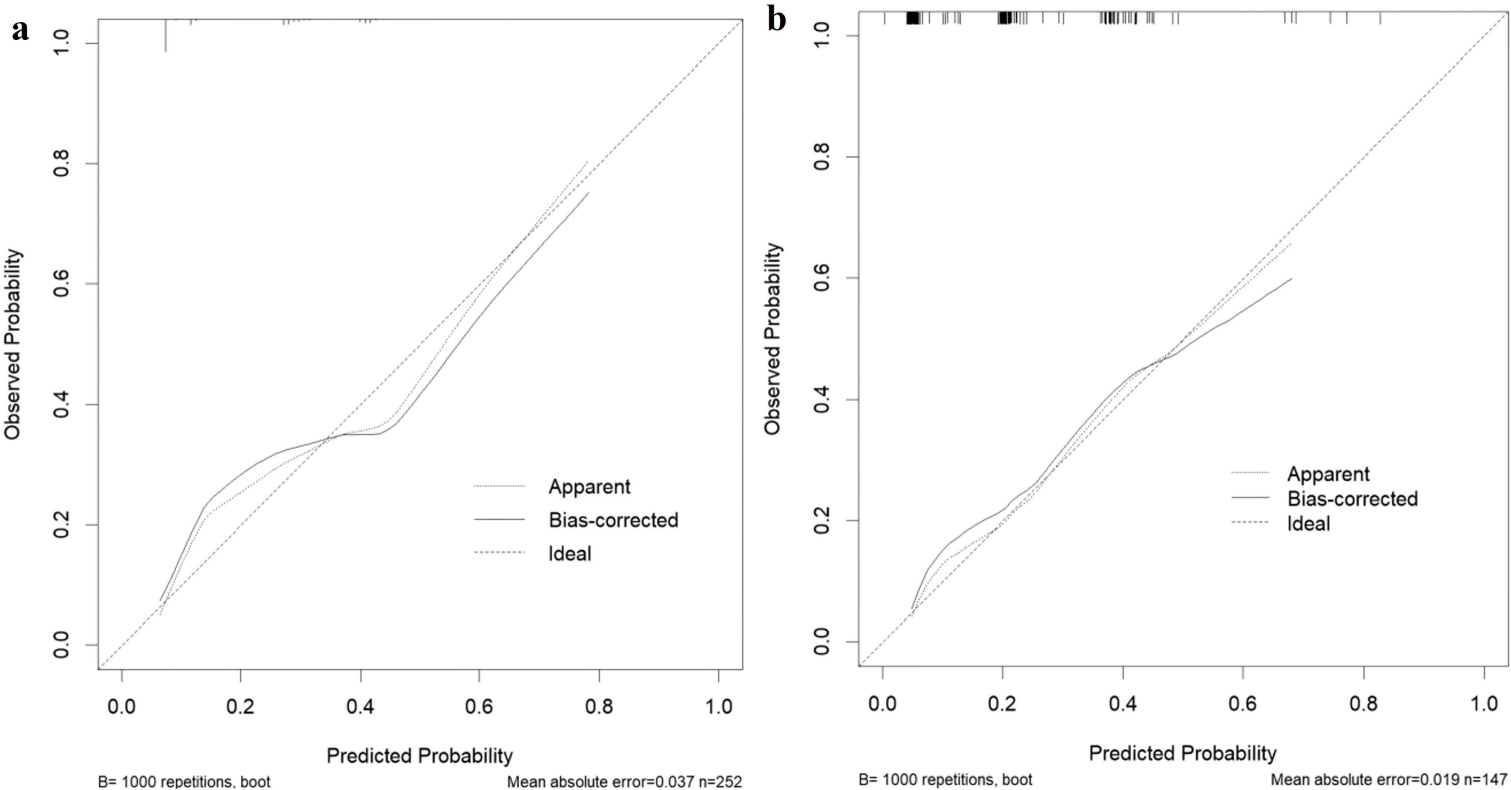
Calibration curve of the clinic-pathological nomogram prediction in the cohort. **(a)** Calibration curve for the training set. **(b)** Calibration curve for the testing set.

Based on the results of a multivariate logistic analysis, four predictive factors were identified—surgical approach, CEA levels, N stage, and perineural invasion—to construct a clinic-pathological nomogram (**Figure 2**). The predictive model demonstrated robust discriminative ability in the training set, with an AUC value of 0.785 (95% CI: 0.725-0.845) (**Figure 3**). Detailed parameters of the model are presented in **Table 3**. In the training set, the model exhibited an accuracy of 73.0%, with sensitivity and specificity values of 71.9% and 73.3%, respectively. The positive predictive value is 44.1%, while the negative predictive value is 89.9%. The calibration curve demonstrates that the model’s predictions align closely with the actual values in the training set (**Figure 4a**). Furthermore, the Hosmer-Lemeshow test produced a P-value of 0.4755, suggesting an acceptable level of calibration.

**Table 3:** Predictive value of the predict model.

Predict model	AUC (95% CI)	ACC	SEN	SPE	PPV	NPV
training set	0.785(0.725-0.845)	0.730	0.719	0.733	0.441	0.899
testing set	0.779(0.696-0.863)	0.721	0.733	0.718	0.400	0.913

**Figure 5:**
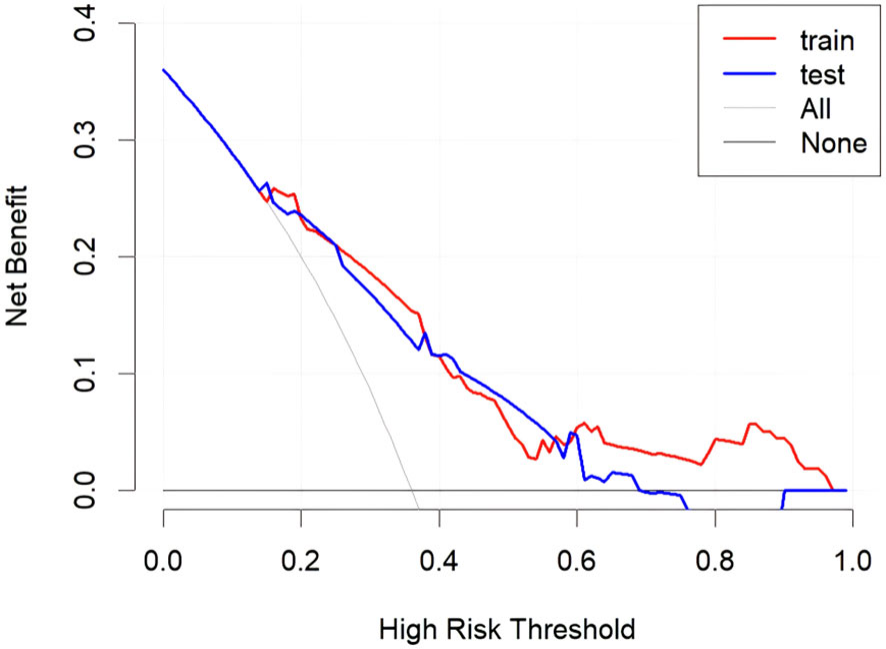
Decision curve analysis for the clinic-pathological nomogram in the training and testing sets.

The DCA for the training set is depicted in **Figure 5**. The DCA indicates that, in certain instances, the application of our constructed nomogram model to predict the risk of lung metastasis in patients who underwent radical surgery for CRC, followed by appropriate intervention, can yield substantial net benefits. Consequently, our nomogram underscores its potential as a valuable tool for clinical decision-making guidance.

### External validation of the predict model

Utilizing patient data from Zhongshan City People’s Hospital as an external validation cohort, the predictive model exhibited satisfactory discriminative performance, evidenced by an AUC value of 0.779 (95% CI: 0.696-0.863). The calibration performance in the test set was also deemed adequate, as indicated by a Hosmer-Lemeshow test P-value of 0.3309 (**Figure 4b**). Furthermore, the Decision Curve Analysis (DCA) demonstrated that the predictive model provided higher net benefits within a specific threshold range in the validation set (**Figure 5**).

### Lung metastasis-free survival analyses

In the training cohort, the median follow-up duration for lung metastasis was 31 months, with a range from 1 month to 80 months. In the test cohort, the median follow-up time was 29 months, spanning from 1 month to 61 months.

**Figure 6:**
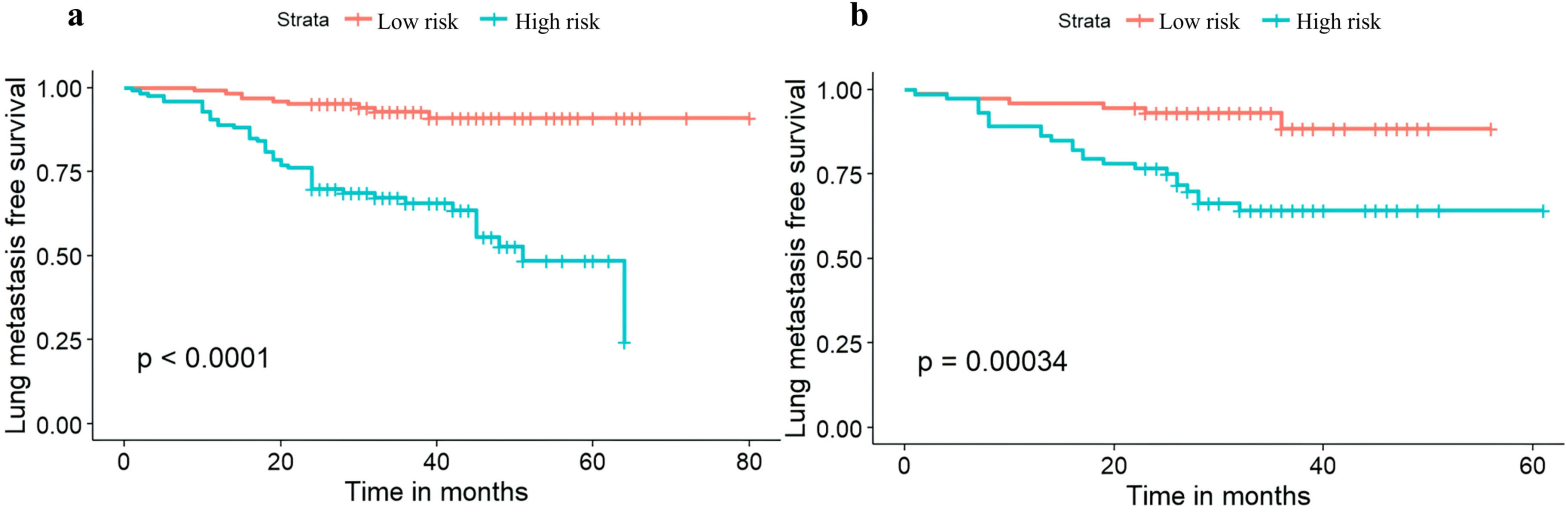
LMFS in high risk and low risk groups. **(a)** LMFS for the Training Set. **(b)** LMFS for the Testing Set.

Furthermore, log-odds scores predicted by logistic regression were associated with LMFS (P <0.001 in the training (a) and testing set (b) respectively) (**Figure 6**).

## Discussion

Colorectal cancer (CRC) is a significant global health issue, being the third most common cancer and the second leading cause of cancer deaths worldwide (1). Although advances in mesenteric-fascial plane surgery have significantly reduced locoregional recurrence, distant metastasis remains the principal cause of mortality in these patients. The lung is the most frequent site of extra-abdominal metastasis (7). According to relevant expert consensus guidelines, various treatment modalities, including surgical resection of metastatic lesions and systemic chemotherapy, can increase the 5-year survival rate to 35-70% in patients with pulmonary metastasis after radical surgery. Particularly for patients with isolated and resect able pulmonary metastasis, active medical intervention can significantly improve the prognosis of this population (21, 22).

Therefore, the prompt detection and aggressive treatment of lung metastases following curative-intent CRC resection are critically important. The construction of predictive models to identify high-risk patients prone to pulmonary metastasis following radical resection can assist surgeon in developing individualized follow-up strategies for different patients, thereby advancing precision medicine.

We developed and externally validated a clinicopathological nomogram to predict the probability of postoperative pulmonary metastasis (PPM) following curative-intent resection of CRC. These variables span surgical, serological, and histological domains, collectively forming a comprehensive risk profile. Each factor captures a distinct aspect of tumor biology, encompassing intrinsic aggressiveness, the systemic host response, and the impact of the surgical approach. Consequently, this predictive model provides greater discriminatory accuracy than algorithms based on a single variable class, such as pathological stage alone. The nomogram translates the multivariable equation into a graphical calculator, facilitating individualized risk quantification at the bedside. In the training cohort (n = 252), the model demonstrated good discrimination and calibration, with a sensitivity of 71.9% and specificity of 73.3%, and a Hosmer-Lemeshow test P-value of 0.475. External validation using an independent cohort (n = 147) confirmed the model’s robust performance, with an area under the curve (AUC) of 0.779, and adequate calibration, with a Hosmer-Lemeshow P-value of 0.331. These results indicate that the nomogram maintains predictive accuracy across diverse patient populations and is suitable for implementation in clinical practice. DCA showed that the nomogram yields a higher net clinical benefit than both the “treat-all” and “treat-none” strategies across a wide range of threshold probabilities. Furthermore, using the median nomogram-derived risk score as the cutoff, patients were divided into high-risk and low-risk cohorts with significantly different lung-metastasis-free survival (LMFS) in both the training and validation cohorts. The median time to pulmonary metastasis was significantly shorter in the high-risk group, highlighting the model’s utility for prognostic stratification and for identifying patients who might benefit from intensified surveillance or adjuvant therapy. By quantifying individual PPM risk, the nomogram facilitates personalized postoperative follow-up strategies. High-risk patients can be enrolled in CT-intensive surveillance programs to facilitate early treatment of oligometastatic disease. In contrast, low-risk patients can be spared unnecessary imaging, thereby optimizing resource allocation and improving cost-effectiveness. Additionally, the nomogram may guide decisions regarding adjuvant therapy. Although adjuvant chemotherapy is standard for stage III CRC, its benefit in stage II disease remains controversial (23). Integrating the nomogram into multidisciplinary discussions could help identify stage II patients with a high cumulative risk who warrant adjuvant therapy, while sparing low-risk patients from unnecessary cytotoxic exposure. In summary, this nomogram provides a practical, evidence-based tool for optimizing both surveillance protocols and adjuvant treatment strategies in patients who have undergone curative resection for CRC.

This study unequivocally demonstrates that preoperative CEA levels serve as an independent risk factor for lung metastasis following R0 resection of CRC. Serum CEA level is involved in intracellular recognition and facilitates adhesion between tumor cells and host cells, which may explain the observed increase in lung metastasis after colorectal cancer surgery (24). It is widely accepted that elevated preoperative CEA levels can facilitate tumor metastasis and postoperative dissemination (25). In this study, CEA measurement was limited to the final preoperative assessment; postoperative levels were not obtained. Consequently, the association between preoperative CEA levels and the development of pulmonary metastasis may not be fully characterized. Future studies should incorporate serial measurements of postoperative CEA to better elucidate the relationship between its dynamics and metastasis. Currently, the tumor-node-metastasis (TNM) classification is a widely endorsed staging system for colorectal cancer. The extent of lymph node involvement has been closely associated with the occurrence of distant metastases (26, 27). During tumor progression, neoplastic cells can disseminate via blood vessels, lymphatic vessels, or nerves (28). Previous research has demonstrated a significant association between perineural invasion and both the malignant pathological phenotype and poor prognosis in CRC. Additionally, perineural invasion has been identified as a prognostic indicator for the efficacy of adjuvant chemotherapy in CRC (29, 30). Given the heterogeneity of molecular subtypes in colorectal cancer patients, it remains challenging to definitively ascertain whether N staging and perineural invasion independently contribute to the development of pulmonary metastasis. In our study, utilizing both univariate and multivariate logistic regression analyses, preoperative CEA level (P = 0.045), N-stage (P<0.001) and perineural invasion (P = 0.047) were identified as independent risk factors for postoperative lung metastasis in colorectal cancer and assigns weighted scores to each. This approach enhances the model’s reliability and credibility.

The protective effect of laparoscopic surgery may stem from several factors, including minimized surgical trauma and reduced postoperative immunosuppression (31). This minimally invasive approach helps preserve host immune function, a key mechanism in controlling occult micro-metastases that may be present at resection. Conversely, open surgery, characterized by larger incisions and more extensive tissue manipulation, can promote tumor cell shedding into the circulation, thereby elevating the risk of distant metastasis. These findings emphasize that the surgical approach is a modifiable risk factor in colorectal cancer management, highlighting the potential of minimally invasive techniques to reduce postoperative recurrence.

The application of machine learning (ML) models for predicting cancer prognosis has garnered growing interest in recent years. Guo et al. recently developed an ML model using the SEER database to predict lung metastasis in patients with CRC (32). The random forest model achieved notably high AUC values: 0.980 in the internal test set and 0.927 in the external validation set. Although the AUC values exceed those of our nomogram, differences in study design must be considered. Specifically, Guo et al. employed a substantially larger dataset (n=39,674) and included a wider array of predictor variables. However, the clinical relevance and interpretability of predictor variables are also critical considerations. The variables in our nomogram were selected via logistic regression and represent commonly used clinical indicators that are readily interpretable to clinicians, enhancing the model’s practical utility. Furthermore, using a more parsimonious set of predictors mitigates the risk of overfitting.

This retrospective study has several inherent limitations. Data extraction from electronic medical records inevitably resulted in missing information and introduced potential selection bias. To mitigate these biases, we implemented stringent inclusion and exclusion criteria, which included the exclusion of patients with incomplete clinical data. Of the 692 patients initially screened, only 399 ultimately met all eligibility criteria and were enrolled in the final analysis. In this process, patients with incomplete follow-up data were likewise excluded, which may have selected for a cohort more adherent to surveillance protocols, potentially biasing the outcomes. Therefore, future prospective controlled trials are needed to validate these findings with greater scientific rigor. As only routinely documented variables were available, we could not incorporate emerging biomarkers such as circulating tumor DNA (ctDNA). Future studies should incorporate a broader range of tumor-related variables, including novel tumor markers, into the model, thereby enhancing both the innovativeness and objectivity of the predictive model. These inherent shortcomings of retrospective analyses preclude definitive causal inferences. The study can only identify associations, and observed relations hips may be influenced by unmeasured confounders. For instance, the higher incidence of pulmonary metastasis following open resection may reflect more advanced disease or unreported surgical comorbidities (e.g., intestinal obstruction or tumor perforation) rather than a direct effect of the surgical approach. These unmeasured confounding factors could bias the statistical outcomes. Furthermore, given the rising adoption of robotic surgery, future studies should analyze it as a distinct cohort to clarify its specific impact.

The two center design with both institutions located in the same region of China, further limits the generalizability of our findings. Before this nomogram can be recommended for widespread clinical use, it is essential to conduct external validation across populations from different regions, as this serves as an effective means of demonstrating the clinical utility of predictive models. Since a number of patients continued their care at other institutions following initial cancer resection, detailed chemotherapy regimens were not available. Consequently, patients could only be stratified as having received “adjuvant chemotherapy” or “no adjuvant chemotherapy.” This lack of granular data on systemic therapy represents a potential unmeasured confounder that may affect the observed incidence of pulmonary metastasis. At the same time, we could not assess whether specific chemotherapeutic regimens differ in their efficacy for preventing pulmonary metastasis. To improve the clinical utility of this model, future validation should include more regional populations.

In our study, the occurrence of pulmonary metastasis was defined as the endpoint event, and patient follow-up was consequently terminated at that point. Consequently, no data were collected on subsequent survival outcomes or treatments received after metastasis. We observed that some patients transferred their care to other institutions following the diagnosis of pulmonary metastasis. Consequently, we were unable to obtain information regarding their treatment regimens following the onset of pulmonary metastasis. Therefore, assessing overall survival alone was deemed methodologically inadequate. We acknowledge that the lack of long-term follow-up data is a major study limitation. Addressing this issue is an explicit goal of our future research. Furthermore, the absence of molecular data constitutes another major limitation. Incorporating biomarkers would likely improve the model’s accuracy. Future predictive algorithms should therefore integrate comprehensive histopathological, molecular, and radiomic data. It should be noted that although some studies suggest that non-contrast chest CT scans alone can diagnose pulmonary metastases from colorectal cancer (21), in our study, the diagnosis of lung metastases was based on contrast-enhanced chest CT or PET-CT to minimize diagnostic errors. However, despite consensus readings by multiple experienced radiologists, diagnostic inaccuracies may still occur.

To address these limitations, future studies should employ a prospective, multicenter design. Prospective data collection would minimize selection bias, ensure more complete follow-up, and facilitate the integration of novel variables such as CT radiomics and liquid biopsy biomarkers. Large, ethnically diverse validation cohorts would provide the statistical power needed to confirm the nomogram’s generalizability across different patient subgroups. Ultimately, broader and richer datasets will facilitate the development of precision medicine tools for risk stratification, enabling tailored surveillance and therapy to improve outcomes for all patients with colorectal cancer.

## Conclusion

We have developed a novel predictive model for lung metastasis in CRC patients following radical surgery. This model integrates clinical data and pathological features, including carcinoembryonic antigen (CEA) levels, surgical approach, N staging, and perineural invasion, into a comprehensive scoring system. This model can assist clinicians in assessing the risk of pulmonary metastasis after radical colorectal cancer surgery, providing a reference for personalized treatment and follow-up. Its clinical intervention effect needs to be further verified by prospective studies.

## Data Availability

The raw data supporting the conclusions of this article will be made available by the authors, without undue reservation.
